# IL-36 Promotes Systemic IFN-I Responses in Severe Forms of Psoriasis

**DOI:** 10.1016/j.jid.2019.08.444

**Published:** 2020-04

**Authors:** Marika Catapano, Marta Vergnano, Marco Romano, Satveer K. Mahil, Siew-Eng Choon, A. David Burden, Helen S. Young, Ian M. Carr, Helen J. Lachmann, Giovanna Lombardi, Catherine H. Smith, Francesca D. Ciccarelli, Jonathan N. Barker, Francesca Capon

**Affiliations:** 1 Department of Medical and Molecular Genetics, School of Basic & Medical Biosciences, King’s College London, London, United Kingdom; 2 Department of Immunobiology, School of Immunology & Microbial Sciences, King’s College London, London, United Kingdom; 3 St John’s Institute of Dermatology, School of Basic & Medical Biosciences, King’s College London, London, United Kingdom; 4 Department of Dermatology, Sultanah Aminah Hospital, Johor Bahru, Malaysia; 5 Department of Dermatology, University of Glasgow, Glasgow, United Kingdom; 6 Division of Musculoskeletal and Dermatological Sciences, University of Manchester, Manchester, United Kingdom; 7 School of Medicine, University of Leeds, Leeds, United Kingdom; 8 National Amyloidosis Centre and Centre for Acute Phase Proteins, Division of Medicine, University College London, London, United Kingdom; 9 Cancer Systems Biology Laboratory, The Francis Crick Institute, London, United Kingdom; 10 School of Cancer & Pharmaceutical Sciences, King’s College London, London, United Kingdom

**Keywords:** CAPS, cryopyrin associated periodic syndrome, CpG, CpG-containing DNA, FDR, false discovery rate, GPP, generalized pustular psoriasis, IL36R, IL-36 receptor, pDC, plasmacytoid dendritic cell, PV, psoriasis vulgaris, TLR- 9, toll-like receptor 9

## Abstract

Psoriasis is an immune-mediated skin disorder associated with severe systemic comorbidities. Whereas IL-36 is a key disease driver, the pathogenic role of this cytokine has mainly been investigated in skin. Thus, its effects on systemic immunity and extracutaneous disease manifestations remain poorly understood. To address this issue, we investigated the consequences of excessive IL-36 activity in circulating immune cells. We initially focused our attention on generalized pustular psoriasis (GPP), a clinical variant associated with pervasive upregulation of IL-36 signaling. By undertaking blood and neutrophil RNA sequencing, we demonstrated that affected individuals display a prominent IFN-I signature, which correlates with abnormal IL-36 activity. We then validated the association between IL-36 deregulation and IFN-I over-expression in patients with severe psoriasis vulgaris (PV). We also found that the activation of IFN-I genes was associated with extracutaneous morbidity, in both GPP and PV. Finally, we undertook mechanistic experiments, demonstrating that IL-36 acts directly on plasmacytoid dendritic cells, where it potentiates toll-like receptor (TLR)-9 activation and IFN-α production. This effect was mediated by the upregulation of *PLSCR1*, a phospholipid scramblase mediating endosomal TLR-9 translocation. These findings identify an IL-36/ IFN-I axis contributing to extracutaneous inflammation in psoriasis.

## Introduction

IL-36α, -β, and -γ (hence IL-36) are group of IL-1 family cytokines that are mainly produced by keratinocytes, monocytes, and dendritic cells (Bassoy et al., 2018). IL-36 signaling plays an important role in epithelial immune homeostasis, and its deregulation has been repeatedly implicated in the pathogenesis of psoriasis vulgaris (PV), a common and chronic, immune-mediated skin disorder (Bassoy et al., 2018).

Numerous studies have shown that IL-36 responses are elevated in PV skin (Mahil et al., 2017, Quaranta et al., 2014, Swindell et al., 2015) where they stimulate chemokine production and amplify the effects of IL-17 signaling (Mahil et al., 2017). Animal studies have also demonstrated that IL-36 promotes the activation of dendritic cells and the polarization of T lymphocytes into T helper type 17 cells (Tortola et al., 2012). Thus, the mechanisms whereby IL-36 contributes to cutaneous inflammation have been extensively investigated. The effects of IL-36 on circulating leukocytes, however, remain poorly understood.

We and others have shown that recessive mutations of the IL-36 receptor antagonist (*IL36RN*) are associated with generalized pustular psoriasis (GPP), a disease variant characterized by severe extracutaneous symptoms (Marrakchi et al., 2011, Onoufriadis et al., 2011). Patients with GPP suffer from flares of skin pustulation that are often accompanied by systemic upset (fever, elevation of acute phase reactants, and neutrophilia) (Burden and Kirby, 2016). This suggests that IL-36 signaling is likely to influence immune responses beyond the skin.

Extracutaneous comorbidities are also well-documented in PV, as individuals suffering from severe disease are at high risk of psoriatic arthritis, metabolic syndrome, and atherosclerosis (Burden and Kirby, 2016, Fang et al., 2016, Shah et al., 2017). Therefore, it has been proposed that PV is a systemic disease, manifesting with skin, joint, and vascular inflammation (Davidovici et al., 2010, Reich, 2012).

In this context, we hypothesized that abnormal IL-36 signaling has extracutaneous effects in both GPP and PV, driving acute systemic flares in the former and contributing to a state of chronic systemic inflammation in the latter. To explore this model, we integrated the transcription profiling of patient leukocytes with ex-vivo IL-36 stimulations. We show that IL-36 potentiates toll-like receptor (TLR) 9 activation and enhances the production of IFN-I, a cytokine that contributes to systemic immunity, arthritis, and atherosclerosis.

## Results

### Expression profiling identifies a IFN-I signature in generalized pustular psoriasis and psoriasis vulgaris whole-blood samples

**Figure 1:**
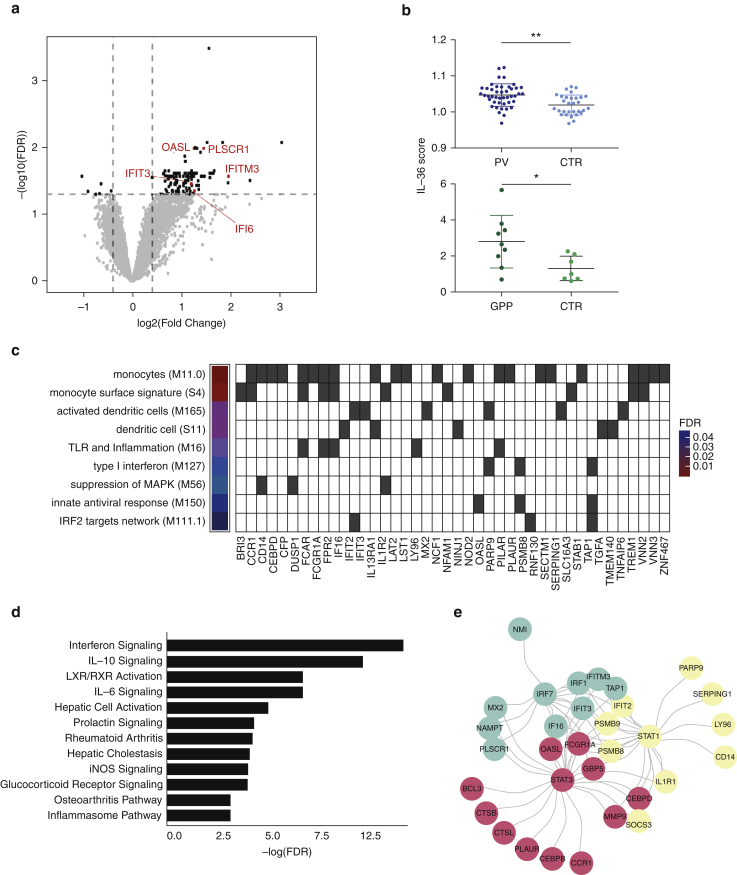

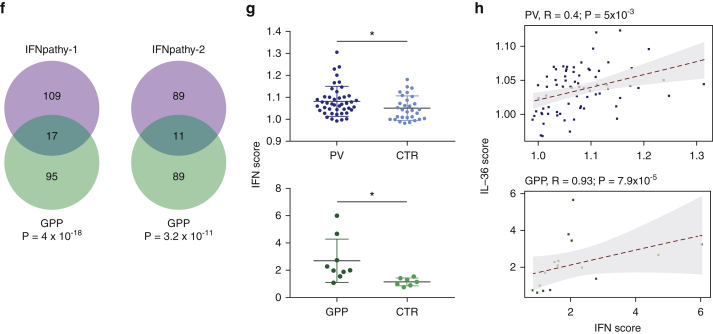
**Transcription profiling of GPP and PV whole blood uncovers a IFN-I signature that correlates with IL-36 activity.** (**a**) Identification of genes that are differentially expressed in GPP. Horizontal and vertical lines represent significance and fold change thresholds, respectively. The genes underlying the IFN score are in red. (**b**) Higher expression of IL-36 dependent genes in whole blood of patients with GPP and PV, compared with controls. (**c**) Transcriptional modules enriched among genes upregulated in GPP. The FDR for each module is reported, with the underlying upregulated genes shown as gray cells. (**d**) Enriched pathways detected among genes over-expressed in GPP. (**e**) Key transcriptional factors driving gene over-expression in GPP. (**f**) Overlap between the genes that are upregulated in GPP and IFNpathies. (**g**) Elevated IFN score in whole-blood samples of patients with GPP and PV, compared with controls. (**h**) IL-36 and IFN scores are significantly correlated, in both patients with GPP and PV. Dashed regression lines are plotted with 95% confidence intervals (gray areas). The data in (b) and (g) are presented as mean ± SD; *P < 0.05, **P < 0.01 (unpaired *t* test). CTR, control; FDR, false discovery rate; GPP, generalized pustular psoriasis; iNOS, inducible nitric oxide synthase; MAPK, mitogen-activated protein kinase; PV, psoriasis vulgaris; SD, standard deviation; TLR, toll-like receptor.

We reasoned that GPP would represent an ideal model in which to investigate the systemic effects of IL-36, because of the well-established link with *IL36RN* mutations (Marrakchi et al., 2011, Onoufriadis et al., 2011) and enhanced IL-36 activity (Johnston et al., 2017). Therefore, we undertook whole-blood RNA sequencing in nine affected individuals and seven healthy controls (Supplementary Table S1). Whereas the deconvolution of transcription profiles showed that leukocyte frequencies were comparable in cases versus controls (Supplementary Table S2), differential expression analysis identified 111 genes that were over-expressed (fold change ≥ 1.5; false discovery rate [FDR] < 0.05) in patients (Figure 1a, Supplementary Table S3). Genes that can be induced by IL-36 (*IL1B, PI3, VNN2, TNFAIP6, and SERPINB1*) were collectively upregulated in cases versus controls (*P* = 0.019) (Figure 1b). Notably, the analysis of a publicly available PV dataset (Wang et al., 2014) identified a moderate, but statistically significant, over-expression of the same genes in patient whole blood (*P* = 0.001) (Figure 1b), suggesting that IL-36 may have systemic effects in PV.

To further explore the biological significance of our findings, we mapped the genes upregulated in GPP to the blood co-expression modules described by Li et al (2014). We found that the over-expressed genes were significantly enriched among modules related to innate immune activation (e.g., enriched in activated dendritic cells, FDR < 0.005) and antiviral responses (e.g., IFN-I response; FDR < 0.05) (Figure 1c). These findings were validated by Ingenuity Pathway Analysis (Qiagen, Aarhus, Denmark), which identified IFN signaling as the most significantly enriched pathway (FDR < 5x10^-6^) (Figure 1d). An upstream regulator analysis also highlighted IRF7, STAT1, and STAT3 as the transcriptional activators that are most strongly associated with gene over-expression (FDR < 10^-10^ for all) (Figure 1e, Supplementary Table S4). Proteins are critical mediators of IFN signal transduction and IFN-α production by plasmacytoid dendritic cells (pDCs) (Honda et al., 2005).

**Supplementary Figure S1:**
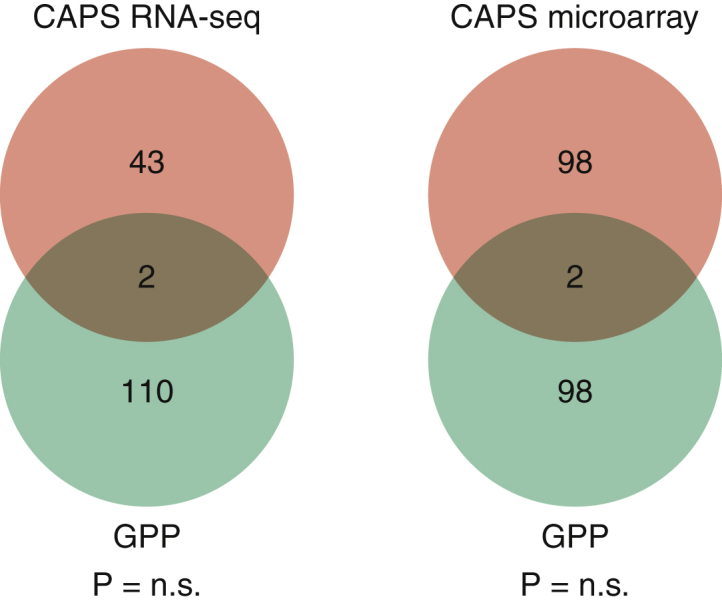
**Lack of evidence for an IFN signature in the leukocytes of patients.** Venn diagrams showing the overlap between the genes that are upregulated in GPP and in the CAPS datasets analyzed by Canna et al. (CAPS-1) and Balow et al. (CAPS-2). Neither overlap was statistically significant. CAPS, cryopyrin associated periodic syndrome; GPP, generalized pustular psoriasis.

Finally, the analysis of two publicly available datasets (Liu et al., 2012, Rodero et al., 2017) demonstrated a significant overlap (*P* < 10^-10^) between the genes that are upregulated in GPP and those that are over-expressed in autoinflammatory syndromes caused by abnormal activation of IFN-I responses (Figure 1f). Notably, no overlap was found with the upregulated genes detected in cryopyrin associated periodic syndrome (CAPS), a disease caused by excessive IL-1 activity, which was analyzed as a negative control (Supplementary Figure S1). Thus, the presence of a IFN-I signature in GPP leukocytes is supported by several lines of evidence.

To further investigate the relevance of these observations, we built an IFN score by measuring the aggregate expression of five genes (*IFI6, IFIT3, IFITM3, OASL, and PLSCR1*), which were upregulated in the GPP dataset and annotated as IFN-I dependent in the Interferome database (Rusinova et al., 2013). The score was elevated in GPP cases compared with controls. A similar increase was observed in the publicly available PV dataset (Figure 1g). We found that the IFN score documented in GPP and PV significantly correlated with the upregulation of IL-36 related genes (*P* < 0.01) (Figure 1h). Thus, we have shown that systemic IFN-I responses are abnormally active in psoriasis, which may be linked to increased IL-36 production.

### IFN-I signature is driven by gene upregulation in neutrophils

The presence of heterogeneous cell populations in whole blood can complicate the interpretation of transcription profiling experiments. Therefore, we, sought to validate our results through an independent analysis of a single cell type. We focused our attention on neutrophils because they play a critical role in systemic inflammation and can be activated by IFN-I (Zimmermann et al., 2016).

**Figure 2:**
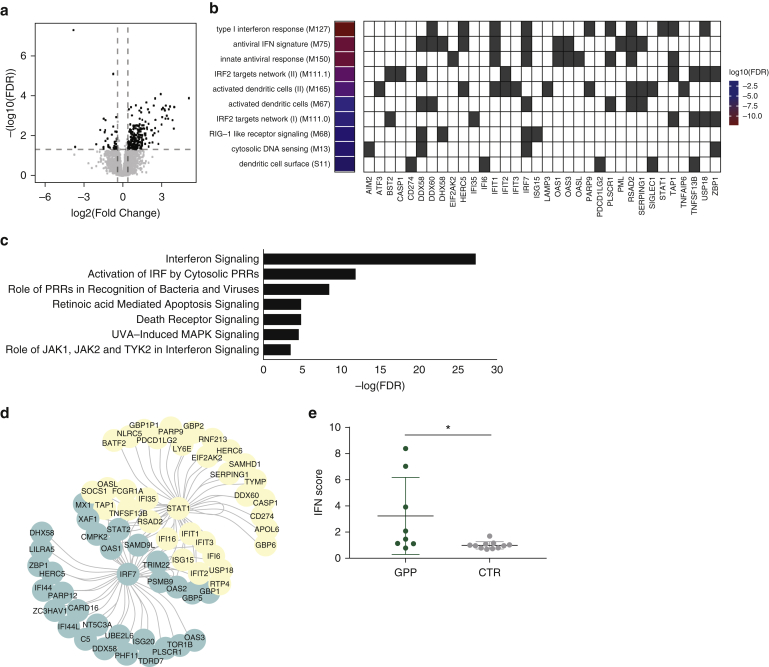
**Transcription profiling of GPP neutrophils confirms the presence of a IFN-I signature.** (**a**) Identification of genes that are differentially expressed in GPP. Horizontal and vertical lines represent significance and fold change thresholds, respectively. (**b**) Transcriptional modules enriched among the genes that are upregulated in GPP. The FDR for each module is reported, with the underlying upregulated genes shown as gray cells. (**c**) Enriched pathways detected among the genes that are upregulated in GPP. IFN-related pathways are highlighted in bold (**d**) Upstream regulator analysis showing that IRF7 and STAT1 drive the upregulation of numerous genes that are over-expressed in GPP. (**e**) Elevated IFN score in the neutrophils of patients with GPP, compared with controls. The data are presented as mean ± SD; *P < 0.05 (unpaired *t* test). FDR, false discovery rate; GPP, generalized pustular psoriasis; PRR, pattern recognition receptors; SD, standard deviation.

We obtained fresh blood samples from 8 GPP cases and 11 controls (Supplementary Table S1). After neutrophil isolation and RNA sequencing, we detected 200 upregulated genes (Figure 2a, Supplementary Table S5). The analysis of transcriptional networks identified IFN-I response as the most significantly enriched module (FDR < 10^-12^), followed by innate antiviral response and antiviral IFN signature (FDR < 10^-10^) (Figure 2b). Ingenuity Pathway Analysis also demonstrated a marked enrichment of pathways related to IFN signaling (FDR < 10^-11^) (Figure 2c) and highlighted IRF7 and STAT1 as the most likely drivers of gene upregulation (FDR < 10^-30^) (Figure 2d, Supplementary Table S6). In keeping with these findings, IFN scores were elevated in GPP cases compared with controls (*P* = 0.02) (Figure 2e). These observations validate the results obtained in whole blood and suggest that the IFN-I signature is driven at least in part, by gene upregulation in neutrophils.

### IFN-I signature can be validated in extended generalized pustular psoriasis and psoriasis vulgaris datasets

We next sought to validate the IFN-I signature through the analysis of further affected individuals.

**Figure 3:**
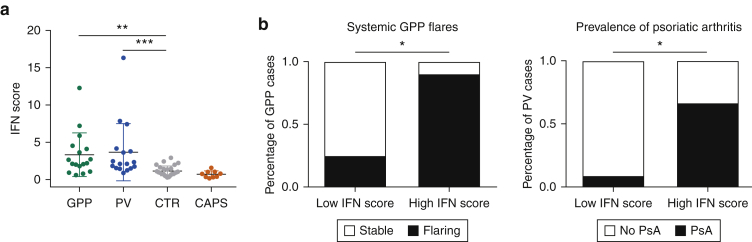
**Validation of the IFN-I signature in extended datasets.** (**a**) Elevated IFN score in the neutrophils of patients with GPP and PV compared with healthy individuals. CAPS cases were analyzed as negative controls. The data are presented as mean ± SD; **P < 0.01 and ***P < 0.001 (1-way analysis of variance followed by Dunnett’s posttest). (**b**) Left: systemic flares are more prevalent in patients with GPP with high IFN scores (n = 8) compared with those with low IFN scores (n = 9). Right: PsA) is more prevalent in patients with PV with high IFN scores (n = 6) compared with those with low IFN scores (n = 11). In both groups, the cut-off between high and low scores was defined as the median +2SD of the values observed in healthy controls. *P < 0.05 (Fisher exact test). CAPS, cryopyrin associated periodic syndrome; GPP, generalized pustular psoriasis; PsA, psoriatic arthritis; PV, psoriasis vulgaris; SD, standard deviation.

We examined neutrophils obtained from 17 GPP cases (including eight newly recruited cases) and 16 patients with PV suffering from severe disease (average Psoriasis Area and Severity Index, 17.9). We also analyzed two control groups, including 9 individuals affected by CAPS and 26 healthy volunteers. Real-time PCR demonstrated that the IFN score was significantly increased in GPP and PV cases compared with healthy controls (*P* < 0.005). Conversely, and in keeping with the specificity of our observations, the scores of patients with CAPS were within the normal range defined in unaffected individuals (Figure 3a).

Notably, medical records showed that patients with GPP with high IFN scores were more likely to experience systemic flares than those with low scores (88% vs. 33%; *P* = 0.049). Likewise, the prevalence of psoriatic arthritis was higher among patients with PV with high IFN scores (67% vs. 18%; *P* = 0.03) (Figure 3b).

Thus, the IFN-I signature detected by RNA sequencing can be validated in independent PV and GPP samples, where it is associated with extracutaneous morbidity.

### IL-36 receptor is expressed on the surface of plasmacytoid dendritic cells

**Figure 4:**
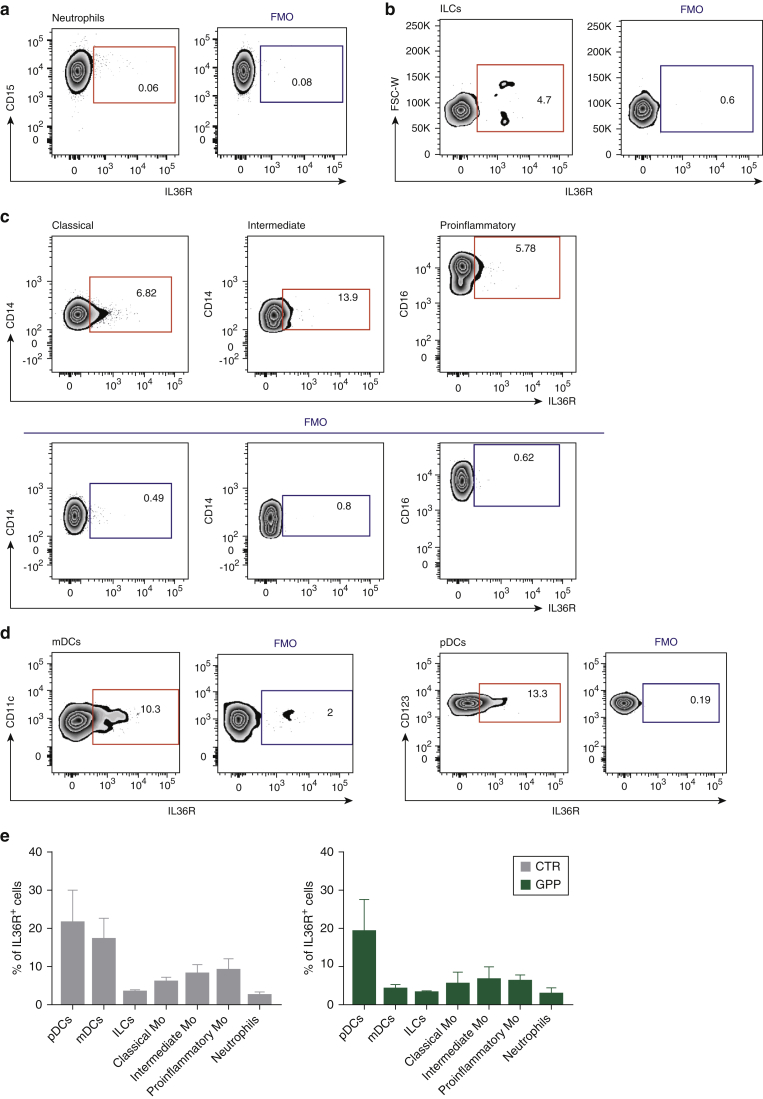
**The IL-36 receptor is preferentially expressed by plasmacytoid dendritic cell s.** (**a–e**) Representative flow cytometry plots showing IL36R surface expression compared with fluorescence minus one control. (**a**) neutrophils (gated as CD14^+^, CD15^+^, CD16^+^ cells); (**b**) innate lymphoid cells (lineage^-^ [CD3^-^, CD4^-^, CD19^-^, CD20^-^, CD56^-^], CD127^+^); (**c**) monocytes (CD3^-^, CD20^-^, CD19^-^, CD56^-^) separated into classical (CD16^-^, CD14^high^), intermediate (CD16^+^, CD14^+^), and pro-inflammatory (CD16^high^, CD14^-^) populations; (**d**) pDCs (lineage^-^, HLA-DR^+^, CD123^+^, CD11c^-^) and mDCs (lineage^-^, HLA-DR^+^, CD123^-^, CD11c^+^). (**e**) Histogram showing the percentage IL36R^+^ cells in each leukocyte population. Data were obtained in at least three GPP cases and three sex-matched controls. Results are presented as mean ± SEM. No significant differences were observed between GPP cases and healthy donors. FMO, fluorescence minus 1; IL36R, IL-36 receptor; GPP, generalized pustular psoriasis; mDC, myeloid dendritic cell; Mo, monocytes; pDC, plasmacytoid dendritic cell; SEM, standard error of the mean.

We next hypothesized that IL-36 has a direct effect on IFN-I producing cells. To investigate this possibility, we systematically examined the surface expression of the IL-36 receptor (IL36R) in innate immune cells. Similar to published findings (Foster et al., 2014), we found that IL36R was barely detectable on the surface of healthy neutrophils (Figure 4a), suggesting that the effects of IL-36 on these cells are mediated by the activation of different immune population(s).

**Supplementary Figure S2:**
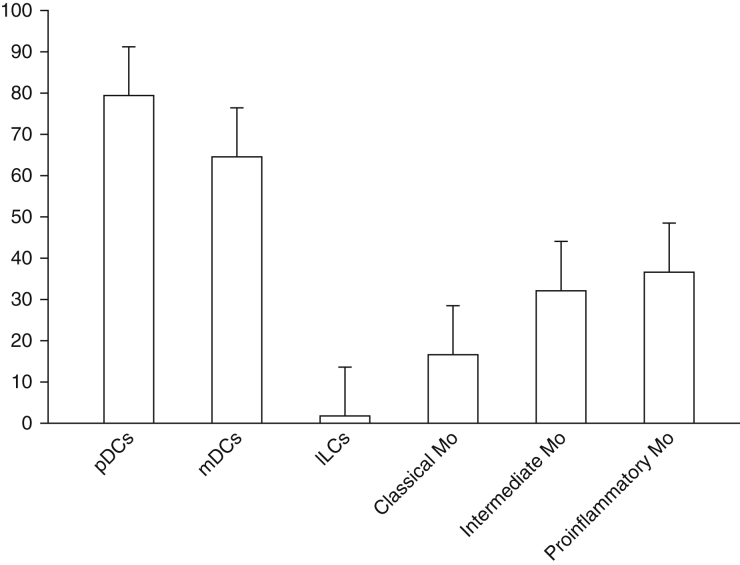
**IL-36 receptor surface expression measured as mean fluorescence intensity.** Data are presented as the mean (± SEM) of measurements obtained in three unrelated healthy donors. ILC, innate lymphoid cell; Mo, monocyte; SEM, standard error of the mean.

We also showed that IL36R^+^ cell numbers were low among innate lymphoid cells (Figure 4b) and in monocytes (Figure 4c). Higher IL36R levels were observed in myeloid dendritic cells and pDCs (Figure 4d, Supplementary Figure S2), with the largest percentage of IL36R^+^ cells detected in the pDCs of patients with GPP (Figure 4e). Thus, we have shown that IL36R is robustly expressed in pDCs, which are the main producers of IFN-α (a member of the IFN-I family) in the immune system.

### IL-36 potentiates IFN-α production in response to toll-like receptor 9 stimulation

**Figure 5:**
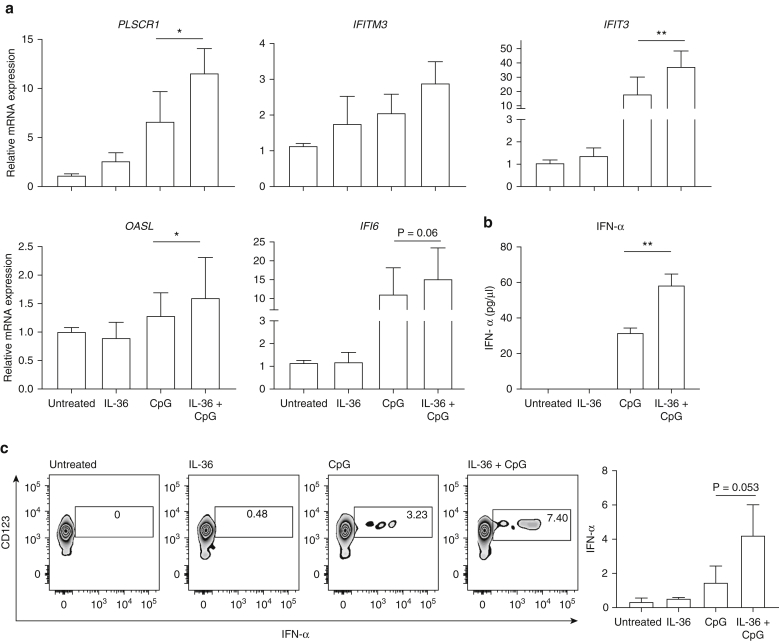
**IL-36 enhances the production of IFN-α downstream of toll-like receptor 9.** (**a**) PBMCs were stimulated with CpG for 6 hours, in the presence or absence of IL-36 pretreatment (6 hours). The expression of IFN signature genes was measured by real-time PCR. Data represent the mean ± SEM of results obtained in three independent donors, each stimulated in triplicate. (**b**) After PBMC stimulation, IFN-α production was measured by ELISA. Data represent the mean ± SEM of results obtained in two independent donors, each stimulated in triplicate. (**c**) After PBMC stimulation, the percentage of IFNα^+^ pDCs was determined by flow cytometry. A representative set of plots is shown (left), together with the data obtained in three independent healthy donors (right). *P < 0.05; **P < 0.01 (Friedman test, with Dunn posttest). CpG, CpG-containing DNA; PBMC, peripheral blood mononuclear cell; pDC, plasmacytoid dendritic cell; SEM, standard error of the mean.

Based on the results obtained in the previously mentioned experiments, we hypothesized that IL-36 potentiates IFN-I production by pDCs. To investigate, we pretreated peripheral blood mononuclear cells obtained from healthy donors with IL-36 or vehicle. We then stimulated the cells with CpG-containing DNA (CpG), a TLR-9 ligand, which induces IFN-α release by pDCs. Finally, we measured the upregulation of the IFN signature genes as a readout of IFN-I production. Whereas CpG increased the expression of most signature genes, its effect was more pronounced in cells that had been preincubated with IL-36 (*P* < 0.05 for *IFIT3*, *OASL*, and *PLSCR1*) (Figure 5a). This observation was validated by direct measurements of IFN-α production, showing increased cytokine release following IL-36 pretreatment (Figure 5b). Finally, flow cytometry documented an increased proportion of IFNα^**+**^ pDCs among the cells that had been stimulated with IL-36 and CpG, compared with those that had been exposed to CpG alone (Figure 5c). Thus, multiple experimental readouts support the notion that IL-36 upregulates TLR-9–dependent IFN-α release.

### IL-36 upregulates *PLSCR1*, a known toll-like receptor 9 transporter

We next sought to define the mechanisms whereby IL-36 enhances cytokine production downstream of TLR-9. A closer inspection of the peripheral blood mononuclear cell stimulation results showed that IL-36 treatment upregulates *PLSCR1*, even in the absence of CpG. The gene encodes phospholipid scramblase 1, a protein that regulates TLR-9 trafficking to the endosomal compartment (Talukder et al., 2012).

**Figure 6:**
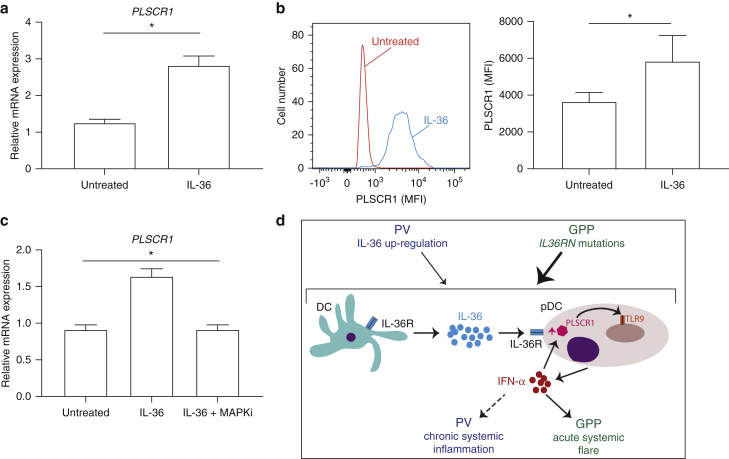
**IL-36 upregulates PLSCR1.** (**a**) After treatment of PBMCs with IL-36, *PLSCR1* expression was measured by real-time PCR. (**b**) After IL-36 treatment of pDCs, PLSCR1 MFI was measured by flow cytometry, in gated PSLCR1^+^ pDCs. A representative histogram is shown on the left. (**c**) After pretreatment with SB203580 (MAPKi), PBMCs were stimulated with IL-36. *PLSCR1* expression was then determined by real-time PCR. (**d**) Proposed pathogenic model. IL-36 produced by mDC upregulates PLSCR1 in pDCs, potentiating TLR-9 dependent IFN-α release. IFN-α induces further *PLSCR1* transcription, thus propagating an inflammatory feed-forward loop. All data are shown as mean ± SEM of results obtained in at least three donors, each stimulated in triplicate. *P < 0.05 (Wilcoxon signed rank test [a, b] and Friedman test with Dunn posttest [c]) mDC, myeloid dendritic cell; MFI, mean fluorescence intensity; PBMC, peripheral blood mononuclear cell; pDC, plasmacytoid dendritic cell; TLR, toll-like receptor; SEM, standard error of the mean.

To further explore the link between IL-36 and PLSCR1, we first validated our initial observation in additional donors (Figure 6a). Next, we demonstrated that IL-36 treatment increases PLSCR1 protein levels in isolated pDCs, showing a direct effect of the cytokine on these cells (*P* < 0.05) (Figure 6b). Finally, we investigated the mechanism whereby IL-36 up regulates *PLSCR1*. As expected for an IFN signature gene, an analysis of the *PLSCR1* promoter uncovered a STAT1 binding site. Because IL-36 can signal through mitogen-activated protein kinases (Bassoy et al., 2018), and that there have been reports of cross-talk between STAT1 and mitogen-activated protein kinase signaling (Zhang et al., 2004), we reasoned that the latter pathway was likely to be involved. Real-time PCR experiments confirmed this hypothesis because the SB-203580 mitogen-activated protein kinase inhibitor abolished the effect of IL-36 on *PLSCR1* expression (Figure 6c).

Thus, we have demonstrated that IL-36 can act directly on pDCs, where it upregulates PLSCR1, in a mitogen-activated protein kinase–dependent fashion.

## Discussion

Whereas PV has been historically described as a dermatological condition, the importance of extracutaneous comorbidities are increasingly recognized (Armstrong et al., 2013). Notably, the prevalence of most comorbid conditions increases with the severity and the duration of the disease (Burden and Kirby, 2016, Egeberg et al., 2017). Therefore, there is a dose-dependent association between cutaneous and extracutaneous inflammation, which suggests a shared systemic pathogenesis. The underlying pathways, however, remain poorly understood.

Here, we demonstrated that IL-36 signaling is enhanced in the leukocytes of patients with PV, where abnormal IL-36 activity correlates with IFN-I over-expression. Whereas many genes that are induced by IL-36 are also upregulated by IL-1, this set of shared targets does not include mediators of IFN-I production (Swindell et al., 2018). Accordingly, we found that IFN signature genes are not over-expressed in CAPS, a condition caused by excessive IL-1 signaling. Thus, IL-1 is unlikely to play a significant role in promoting IFN-I responses in psoriasis.

Several studies have found that IFN-I is a mediator of vascular inflammation, which promotes the recruitment of leukocytes to atherosclerotic plaques (Goossens et al., 2010, Niessner et al., 2007). Experiments carried out in animal models have also shown that TLR-9–dependent IFN-I production is a key driver of systemic autoimmunity (Di Domizio et al., 2012).

In keeping with these observations, signatures of excessive IFN-I activity have been documented in various diseases presenting with prominent systemic involvement. One example is systemic lupus erythematosus, a disorder associated with skin and joint inflammation, accelerated atherosclerosis, and upregulation of genes such as *IFI6* and *OASL* (El-Sherbiny et al., 2018). Three independent studies have reported that IL-36 serum levels correlate with disease activity in systemic lupus erythematosus (Chu et al., 2015, Ismail et al., 2018, Mai et al., 2018), which further reinforces the link between IL-36 and IFN-I. Our work adds to these observations and provides mechanistic insights into the underlying pathways.

Our computational and experimental results implicate pDCs as the most likely mediators of IL-36 activity. First, we identified IRF7 as one of the most significant drivers of differential gene expression in GPP. Second, we demonstrated that IL36R levels are highest in pDCs, especially among patients with GPP. Notably, it has long been established that pDCs accumulate within psoriatic skin lesions, where they contribute to early disease processes alongside slanDC (Hänsel et al., 2011, Nestle et al., 2005). It has also been reported that IL36R is abundantly expressed in various classes of skin-resident dendritic cells (Dietrich et al., 2016). Thus, it is tempting to speculate that IL-36 mediated pDC activation may also have a pathogenic role in skin.

Our results show that the effects of IL-36 on pDCs are mediated, at least in part, by PLSCR1 upregulation. A *PLSCR1* small interfering RNA knockout inhibits IFN-I production by human pDCs (Talukder et al., 2012), so it is reasonable to hypothesize that an increase in gene expression would have the opposite effect. Whereas the PLSCR1 induction observed in our IL-36 stimulation experiments was modest (1.5-2.0 fold), it might be sufficient to activate a feed-forward loop whereby upregulated PLSCR1 promotes the production of IFN-I, which in turn induces further *PLSCR1* transcription. Self-amplifying loops are a key feature of IFN-I signaling because they are required for robust antiviral responses (Hall and Rosen, 2010).

We cannot exclude the possibility that additional IL-36 responsive genes or cell types may also contribute to the upregulation of IFN-I. However, we have found that IL-36 does not affect the expression of *TLR9* or that of key downstream genes (*IRF1*, *IRF3*, *IRF7*; data not shown). We have also observed that genes driving other antiviral pathways (*DDX58*/RIG-I, *IFIH1*/MDA5, *TMEM173*/STING) are not upregulated in PV or GPP whole blood.

Whereas our pDC stimulations were carried out with a synthetic TLR-9 agonist, the identity of the agents that cause IFN-α production in patients remains to be determined. In lesional skin, pDCs are activated by self-nucleic acids released by apoptotic keratinocytes and bound to the LL-37 antimicrobial peptide (Lande et al., 2007). Our transcriptomic data, however, suggests that this mechanism is unlikely to be relevant at the systemic level. Whereas *CAMP* (the gene encoding LL-37) was upregulated in psoriatic skin, it was not over-expressed in GPP or PV whole blood. Moreover, there was no correlation between *CAMP* whole-blood expression and the upregulation of IFN-I genes (*r* < 0.1). Thus, the agents that activate the circulating pDC of patients with psoriasis may be different from those that are present in skin.

In conclusion, we have identified an IL-36/TLR-9 axis which upregulates systemic IFN-I production in psoriasis (Figure 6d). In patients with GPP, the effects of IL-36 signaling are amplified by inherited *IL36RN* mutations, a phenomenon which is likely to account for the severe nature of systemic flares. In PV, the T helper type 17-dependent upregulation of IL-36 cytokines is associated with a less pronounced transcriptional signature and with signs of chronic systemic inflammation.

Because IL-36 is down-regulated by IL-17 inhibitors, such as secukinumab (Kolbinger et al., 2017), it is possible that treatment of psoriasis with IL-17 antagonists might also modulate IFN-I production. Notably, the effects of direct IL-36 inhibition are currently being investigated in clinical trials, with promising results obtained in a phase I study (Bachelez, 2018). In this context, our work suggests that IL-36 antagonists have the potential to improve systemic IFN-I upregulation and extracutaneous manifestations of psoriasis.

## Methods

### Human subjects

The study was performed according to the principles of the Declaration of Helsinki. Patients were ascertained at St John’s Institute of Dermatology and Royal Free Hospital (London, United Kingdom), Glasgow Western Infirmary (Glasgow, United Kingdom), Salford Royal Foundation Trust (Manchester, United Kingdom), and Hospital Sultanah Aminah (Johor Bahru, Malaysia). The study was approved by the ethics committees of participating institutions, and written informed consent was obtained from all participants.

Nine unrelated patients with GPP and seven healthy controls were recruited for whole-blood RNA sequencing, whereas neutrophil RNA sequencing was carried out in 8 patients with GPP and 11 healthy controls. Five controls and six cases were common to both studies (Supplementary Table S1). For the validation of neutrophil RNA sequencing results, fresh blood was obtained from 17 GPP, 26 control, 9 CAPS, and 17 PV individuals (Supplementary Table S7). All patients with PV suffered moderate-to-severe disease (Psoriasis Area Severity Index > 10) and were recruited from the same center (severe psoriasis service, St John’s Institute of Dermatology). Patients presenting with joint pain were referred to an expert rheumatologist, who diagnosed psoriatic arthritis, when applicable. The *IL36RN* gene was screened in all GPP cases and mutations were identified in four individuals (Supplementary Table S1).

### RNA sequencing data analysis

The raw sequence data generated in house and that retrieved from public repositories (Supplementary Table S8) were processed with the same computational pipeline (described in Supplementary Materials) to standardize the data analysis process. Genes were considered upregulated if the fold change exceeded 1.5 (FDR < 0.05). When RNA sequencing and microarray data were compared, the analysis focused on the 100 genes that were most significantly upregulated in each sample to account for the different sensitivity of the two platforms.

Genes upregulated in GPP were used as input for pathway and upstream regulator enrichment analyses (Ingenuity Pathway Analysis). STAT1-, STAT3-, and IRF7-centered networks were visualized with the igraph version 1.0.1 R package.

The transcriptional modules that were active in our datasets were selected from the library published by Li et al (2014). The enrichment test function was then applied to the lists of upregulated genes.

The IFN score was built using the five IFN-I dependent genes that were most upregulated in GPP whole blood (*PLSCR1, OALS, IFI6, IFIT3, and IFITM3)*. Because IL-36 dependent genes have not been systematically characterized in leukocytes, the IL-36 score was based on the analysis of five genes, which were strongly induced by IL-36 in keratinocytes (Mahil et al., 2017) and robustly expressed in whole blood (*IL1B, PI3, VNN2, TNFAIP6, and SERPINB1)*. Both scores were derived by normalizing reads per kilo base per million mapped reads values to a calibrator sample and then computing the median expression of the five genes.

### Statistics

Differences between patient and control cytokine scores were assessed using an unpaired *t* test or 1-way analysis of variance, as appropriate. To account for donor variability in cytokine responses, IL-36/CpG stimulations were analyzed with non-parametric methods (Wilcoxon signed rank test for comparisons between two groups and Friedman test for comparison between three groups) because these do not assume equal variance among samples. The correlation between cytokine scores was calculated using Spearman method. The significance of overlaps observed in Venn diagrams was computed with a hyper-geometric test and confirmed by bootstrap analysis. Fisher exact test was used to compare the clinical features of patients with high and low IFN scores.

### Data availability statement

According to UK research councils’ Common Principles on Data Policy, the RNA sequencing data generated in this study are available through the Gene Expression Omnibus (identifier: GSE123787).

## ORCIDs

Marika Catapano: https://orcid.org/0000-0003-2344-6067

Marta Vergnano: https://orcid.org/0000-0003-4654-5519

Marco Romano: https://orcid.org/0000-0001-6089-5828

Satveer. K. Mahil: https://orcid.org/0000-0003-4692-3794

Siew-Eng Choon: https://orcid.org/0000-0002-7796-5746

A. David Burden: https://orcid.org/0000-0001-7395-9931

Helen S. Young: https://orcid.org/0000-0003-1538-445X

Ian M. Carr: https://orcid.org/0000-0001-9544-1068

Helen J. Lachmann: https://orcid.org/0000-0001-8378-2498

Giovanna Lombardi: https://orcid.org/0000-0003-4496-3215

Catherine H. Smith: https://orcid.org/0000-0001-9918-1144

Francesca D. Ciccarelli: https://orcid.org/0000-0002-9325-0900

Jonathan N. Barker: https://orcid.org/0000-0002-9030-183X

Francesca Capon: https://orcid.org/0000-0003-2432-5793

## Conflict of Interest

The authors have received funding or fees from Abbvie and Novartis (CHS, HSY, JNB); Almirall, Jansen, Leo Pharma, and UCB (HSY, JNB); AnaptysBio (FC); Aspire Pharma, Johnson and Johnson, MEDA Pharmaceuticals (HSY); Boehringer Ingelheim (FC, JNB, ADB); Bristol Myers Squibb, Celegene, Ely Lily, Pfizer, Samsung, Sienna, Sun Pharma (JNB); GSK, Roche, Regeneron, Sanofi (CHS).

## Figures and Tables

**Supplementary Table S7:** Details of Individuals Analyzed for IFN Score Validation

Group	Individuals with Active Disease	Sex	Average Age, y	Treatment
CAPS	4/9	3M, 6F	44.6	cankinumab (n = 7), anakinra (n = 2)
GPP	11/17	2M, 15F	56.5	adalimumab (n = 3), certolizumab (n = 1), infliximab (n = 2), ustekinumab (n = 1), acitretin (n = 1), ciclosporin (n = 2), MTX (n = 1), prednisolone (n = 1), topical treatment (n = 4), no treatment (n = 1)
PV	17/17	13M, 4F	42.5	apremilast (n = 1), etanercept (n = 1), infliximab (n = 1), ixekizumab (n = 1), secukinumab (n = 1), ustekinumab (n = 4), MTX (n = 1), topical treatment (n = 6), PUVA (n = 1)
Control	NA	3M, 23F	46.4	NA

Abbreviations: CAPS, cryopyrin associated periodic syndrome; F, female; GPP, generalized pustular psoriasis; M, male; NA, not applicable; PV, psoriasis vulgaris; MTX, Methotrexate

The percentage of patients receiving systemic treatment was comparable in the PV-only and PV with psoriatic arthritis groups.

**Supplementary Table S8:** Details of Publicly Available Datasets

Dataset	Disease	Ethnicity	Tissue	Platform	Identifier
CAPS-1	NOMID	European	Whole blood	RNAseq	GSE57253
CAPS-2	CAPS	European	Whole blood	microarray	Suppl. Material, PMID: 23223423
IFNpathy-1	IFN-I mediated autoinflammation	European	Whole blood	RNAseq	E-MTAB-5735
IFNpathy-2	CANDLE	European	Whole blood	microarray	data provided by authors
PV	Psoriasis vulgaris	European	Whole blood	RNAseq	GSE67785

All patients had active disease. Raw data were downloaded from the Gene Expression Omnibus as CEL (CAPS-2 and IFN-pathy2) or FASTQ files. The former was analyzed with the limma package, whereas the latter were processed with the standardized pipeline described in Supplementary Materials and Methods).

**Supplementary Table S9:** Flow Cytometry Antibodies

Target	Dilution	Cat number	Fluorochrome	Supplier
CD16	1:20	48-0168-42	Efluor450	ThermoFisher
	1:22	130-098-101	APC	Miltenyi Biotec
CD56	1:33	318316	Alexa Fluor 700	BioLegend
CD19	1:20	302242	BV510	BioLegend
	1:22	555412	FITC	BD
CD20	1:33	130-096-649	PE-Cy7	Miltenyi Biotec
CD14	1:20	555398	PE	BD
CD3	1:33	317306	FITC	BioLegend
	1:22	130-098-162	FITC	Miltenyi Biotec
CD127	1:20	351320	PE-Cy7	BioLegend
HLA-DR	1:33	307636	BV421	BioLegend
CD11c	1:20	301638	BV650	BioLegend
CD123	1:30	306030	BV711	BioLegend
Lineage marker^a^	1:10	B29559	PE	Beckman Coulter
CD15	1:33	301904	FITC	BioLegend
	1:22	130-098-010	PE	Miltenyi Biotec
IL36R	1:10	BAF	Streptavidin	BD
Streptavidin^b^	1:100	405207	APC	BioLegend
CD45	1:22	130-098-139	VioBlue	Miltenyi Biotec
CD24	1:22	130-099-935	APC Vio770	Miltenyi Biotec
PLSCR1	1:50	ab180518	Rabbit-IgG	Abcam
Rabbit IgG^c^	1:100	406416	Alexa Fluor 488	BioLegend
IFN-α	1:10	130-092-602	APC	Miltenyi Biotec

a CD3/CD14/CD19/CD20/CD56.

b Target of secondary biotinylated antibody used for IL36R detection.

c Target of secondary antibody used for PLSCR1 detection.

## Supplementary Materials and Methods

### RNA sequencing

Total RNA was isolated from whole blood collected in Tempus Blood RNA Tube using a Tempus Spin RNA Isolation Kit (ThermoFisher Scientific, Waltham, MA). Samples were subjected to globin depletion using a GLOBINclear Kit (ThermoFisher Scientific). Neutrophil RNA was isolated with GeneJET RNA Purification Kit (ThermoFisher Scientific).

Whole-blood RNA was sequenced on a HiSeq 3000 Illumina platform (Illumina, San Diego, CA), obtaining 150 base pair paired-end reads. Neutrophil RNA was sequenced on a NextSeq 500 Illumina platform obtaining 75 base pair single-end reads. The quality of the sequence data was assessed using FastQC (Illumina). Alignment against the HG38 human genome was implemented in TopHat (Kim et al., 2013) with indexes generated by Botwie2. Read counts, produced by HTseq-count, were used as input for the differential expression analysis, which was performed with DESeq2 (Love et al., 2014) (R package, version 16.2), using sex as a co-variate.

### Cell isolation and culture

Neutrophils were purified using the MACSxpress Whole Blood Neutrophil Isolation Kit (Miltenyi Biotec, Bergisch Gladbach, Germany). Peripheral blood mononuclear cells (PBMCs) were isolated using Ficoll-Paque PLUS (GE Healthcare, Little Chalfont, United Kingdom). Plasmacytoid dendritic cells were purified from PBMCs using a Plasmacytoid Dendritic Cell Isolation Kit (Miltenyi Biotec). PBMCs and Plasmacytoid dendritic cells were cultured at a density of 2.5 × 10^6^ cells/ml and 2.5 × 10^5^ cells/ml, respectively, in RPMI Glutamax (Gibco, Waltham, MA) supplemented with 10% fetal bovine serum and 1% penicillin-streptomycin. Cells were stimulated with 50 ng/ml IL-36α (Bio-Techne, Minneapolis, MN) for 6 hours and with 1.6 ng/ml ODN-A CpG (InvivoGen, San Diego, CA) for another6 hours. For IFN-α and PLSCR1 flow cytometry analysis, Brefeldin A (BioLegend, San Diego, CA) was added to the stimulated cells at a 1:1,000 dilution after 9 hours. Response to stimulation was measured by real-time PCR, ELISA, or flow cytometry.

### Real-time PCR and ELISA

RNA samples were isolated with the GeneJET RNA Purification Kit (ThermoFisher Scientific). Following reverse transcription with the nanoScript2 kit (PrimerDesign, Southampton, United Kingdom), gene expression was assessed by real-time PCR using PrecisionPLUS Master Mix with SYBR and ROX (PrimerDesign) in conjunction with the following primer pairs:

*IFI6*: 5′-TTTCTTACCTGCCTCCACCC-3′; 5′-CCATCTATCAGCAGGCTCCG-3′

*IFIT3*: 5′-TTGGTGACCTCACTCATGATGG-3′; 5′-GCACAGACCTAACAGCACCC-3′

*IFITM3*: 5′-CACTGGGATGACGATGAGCA-3′; 5′-TCGCCTACTCCGTGAAGTCTA-3′;

*OASL*: 5′-GGAACCTGGAAGGACAGACG-3′; 5′-GTACCAGCAGAGGGCACG-3′

*PLSCR1*: 5′-AGGAGGATACCCAACTGGCA-3′; 5′-CGGCAGCCAGAGAACTGTTTTA-3′

*IL1B:* 5′-GCCCTAAACAGATGAAGTGCTC-3′; 5′-GAACCAGCATCTTCCTCAG-3′.

The IFN score was derived by computing the median relative quantification of the five signature genes, (*PLSCR1, OALS, IFI6, IFIT3, and IFITM3)* according to the method described by Rice et al. (2013). The production of IFN-α was measured using the Human IFN-alpha ELISA kit (Bio-Techne). For whole-blood and PBMC samples, transcript levels were normalized to *B2M* expression, whereas *RPL13A* was used for neutrophils.

### Flow cytometry

The purity of neutrophil isolated for RNA sequencing was measured by staining cells with anti-CD45, anti-CD15, anti-CD16, anti-CD3, anti-CD24, and anti-CD19 antibodies. IL-36 receptor surface expression and IFN-α levels were measured by staining PBMCs with LIVE/DEAD Fixable Near-IR (Invitrogen, Waltham, MA), Fc and monocyte blocker (BioLegend), antibody against the protein of interest and an antibody cocktail for monocytes (anti-CD3, anti-CD20, anti-CD19, anti-CD16, anti-CD14, anti-CD56) or dentritic cells and innate lymphoid cells (lineage, HLA-DR, CD123, CD11c, and CD127). IL-36 receptor expression on neutrophils was determined by staining for the receptor as well as CD15, CD16, and CD14. PLSCR1 expression was measured by staining purified plasmacytoid dendritic cell with anti-CD123, anti-HLA-DR, anti-CD11c, and anti-PLSCR1. Cells were acquired on a BD Fortessa LSR or a BD FACSCanto II instrument (Becton Dickinson, Franklin Lakes, NJ). All data was analyzed using FlowJo version 10 software (Becton Dickinson). The details of all antibodies are reported in Supplementary Table S9.

## Supplementary Data

Supplementary Table S1 Clinical and demographic features of patients with GPP Supplementary Table S2. Frequency of immune populations derived by deconvolution of RNA sequencing profiles. Supplementary Table S3. Genes differentially expressed in GPP whole blood. Supplementary Table S4. Upstream regulator enrichment among genes over[HYPHEN]expressed in GPP whole blood. Supplementary Table S5. Genes differentially expressed in GPP neutrophils. Supplementary Table S6. Upstream regulator enrichment among genes over-expressed in GPP neutrophils.
